# Oral Lesions Induced by Chronic Khat Use Consist Essentially of Thickened Hyperkeratinized Epithelium

**DOI:** 10.1155/2015/104812

**Published:** 2015-09-27

**Authors:** Ochiba Mohammed Lukandu, Lionel Sang Koech, Paul Ngugi Kiarie

**Affiliations:** ^1^Department of Maxillofacial Surgery, Oral Medicine, Pathology and Radiology, School of Dentistry, Moi University, P.O. Box 4606, Eldoret 30100, Kenya; ^2^Department of Oral Biology, Anatomy, Physiology and Biochemistry, School of Dentistry, Moi University, P.O. Box 4606, Eldoret 30100, Kenya

## Abstract

*Objectives.* The habit of khat chewing is prevalent in many Middle Eastern and African cultures and has been associated with various adverse conditions in humans. This study aimed to describe histological changes induced by chronic khat chewing on the buccal mucosa. *Methods.* Biopsies of the buccal mucosa from 14 chronic khat chewers, 20 chronic khat chewers who also smoked tobacco, and 8 nonchewers were compared for epithelial thickness, degree and type of keratinization, and connective tissue changes. *Results.* Tissues from khat chewers depicted abnormal keratinization of the superficial cell layer and showed increased epithelial thickness affecting all layers. Epithelial thickness in control samples was 205 ± 26 *μ*m whereas thickness in khat chewers and khat chewers who smoked tobacco was significantly higher measuring 330 ± 35 *μ*m and 335 ± 19 *μ*m, respectively. Tissues from khat chewers also showed increased intracellular edema, increased melanin pigment deposits, and increased number of rete pegs most of which were thin and deep. *Conclusions.* These results show that oral lesions induced by chronic chewing of khat in the buccal mucosa present with white and brown discoloration due to increased epithelial thickness, increased keratinization, and melanin deposition.

## 1. Introduction

Khat is an evergreen plant grown in the regions around the horn of Africa and the Southern Arabian Peninsula. Fresh leaves and shoots of the khat plant contain an alkaloid chemical known as cathinone which has psychoactive effects comparable to amphetamine. Since the 13th century [1], populations in these regions have engaged in the habit of chewing khat leaves and shoots as a mood altering drug. In countries such as Yemen, up to 90% of adult males and more than half of adult females are estimated to chew khat for between three and four hours daily [1, 2]. Currently, the habit is spreading to other parts of the world where users are predominantly immigrants from countries where khat use is widespread [2].

The habit of chewing khat is associated with adverse effects in various body systems [3]. It has been reported through evidence from animal studies that khat decreases the systemic capacity of the body to handle reactive oxygen species [4] and therefore has potential to cause damage to cells and tissues. During chewing sessions, large amounts of khat leaves, shoots, and barks are placed in the oral cavity and chewed while being kept in the vestibule in close contact with the buccal mucosa [5]. The khat bolus is chewed gradually and continuously for 2 to 10 hours. On average, 100–500 g of khat is chewed by chronic users per day [6]. Over 90 percent of the alkaloid content of khat is extracted into saliva during chewing and most of it is absorbed through the oral mucosa [7]. Therefore, oral tissues, especially the oral mucosa, are exposed to high doses of khat constituents during khat chewing rendering them susceptible to its potentially toxic effects.

Previous studies have reported various detrimental effects of khat on oral tissues [8]. These effects include various forms of periodontal disease, mucosal pigmentation, dental caries, tooth wear, and dental staining [9]. Khat is genotoxic to cells of the oral mucosa [10] and has been associated with oral keratotic white lesions which occur in the same region within the vestibule or buccal mucosa where the khat bolus is placed while chewing [11–13]. Some of these lesions have been reported to show histopathological changes like acanthosis, hyperkeratosis, and mild dysplasia [11]. According to some previous studies, the risk for developing these lesions is especially high among khat chewers who also use tobacco products [14]. In another study, khat chewing was found to be a risk factor for developing cellular atypia, in addition to hyperkeratosis and subepithelial infiltration by chronic inflammatory cells [15].

Even though some studies have found a higher incidence of head and neck cancer in khat chewers compared to nonchewers [16, 17], lesions induced by khat have not been considered potentially malignant [18, 19]. Due to the relatively small number of studies on khat [20] and the methodological weaknesses of studies already carried out [19, 21], there is currently no consensus as to whether khat chewing is a potential risk factor for development of oral cancer [21]. A useful point to start in understanding this potential risk would be to have a detailed clinical and microscopic analysis of oral white lesions induced by chronic khat use. This study therefore sought to describe histopathological features induced by khat when used alone and when used alongside tobacco within the oral mucosa of the chronic khat chewers.

## 2. Materials and Methods

### 2.1. Study Subjects

The use of human subjects in this study was reviewed and approved by the regional Institutional Research and Ethics Committee (IREC) (approval number 000985). A public call by study assistants for volunteers to participate in the study was made in Eldoret and Meru towns of Kenya, and those willing to participate were requested to visit specified dental clinics for screening. The study was conducted on 42 volunteers who met the inclusion criteria for the study and for biopsy procedures. All participants were informed of the purpose of the study and were requested to sign consent forms.

Those included in the study as cases were khat chewers who had used khat for more than 5 years (chronic chewers) and who also had clinically detectable pathological oral white lesions based on common protocol/criteria (Table 1). All participants who eventually participated in the study were male, even though this was not a requirement. The study subjects were divided into three groups: (1) a group of 8 volunteers who were neither tobacco smokers nor khat chewers, (2) a study group of 14 volunteers who were chronic khat chewers but nonsmokers, and (3) a second study group of 20 volunteers who were both chronic khat chewers and tobacco smokers. The first group was the control group and consisted of clinically healthy adult male volunteers undergoing surgical removal of wisdom teeth.

### 2.2. Clinical Procedures

All participants were first subjected to a short interviewer administered questionnaire designed to collect biographic data and information related to khat use, tobacco use, and alcohol drinking. The participants were then subjected to a clinical oral examination on a dental chair with adequate lighting with specific focus on the appearance of their oral mucosa. Clinical images of the buccal mucosa showing the oral lesions were taken prior to biopsy procedures. Control patients were requested to participate in the study by allowing a small piece of buccal mucosal tissue to be biopsied from the incision line during surgical removal of impacted wisdom teeth. For khat chewers, the biopsies were taken from the buccal mucosa at specifically selected sites that were deemed most affected by the habit of khat chewing. The predesigned criteria (Table 1) for oral lesions induced by chronic khat use were used to ensure consistency in the choice of the most affected sites for the purpose of biopsy.

Local anesthesia was achieved through a long buccal nerve block on the side identified for the biopsy. Care was taken to administer the injection away from the site of the biopsy to avoid damage to the tissue. Tissue at the biopsy site was secured and gently retracted using a suture with only one loose knot. An ovoid tissue specimen of about 4 by 8 mm was then excised using a surgical blade. All biopsy specimens were immediately placed in adequate amounts of 4% buffered formalin (pH 7.2) and forwarded to the laboratory for histopathological assessment. Bleeding was controlled by applying pressure and the same suture was then used to stitch and close the wound at the site of the biopsy.

### 2.3. Tissue Preparation, Staining, and Histopathological Assessment

The tissues were fixed for 48 h at room temperature in 4% buffered formalin. Biopsy specimens were grossed and taken through routine procedures for formalin fixed paraffin embedded preparations. Briefly, the formalin fixed tissues were washed in more formalin solution to remove any loose debris and then dehydrated through a series of graded alcohol solutions (70%, 90%, 100%, 100%, and 100% ethanol 1 h each) and then through three changes of xylene for 1 h each. The tissues were then incubated overnight at 65°C in Paraplast Plus paraffin (Thermo Fisher Scientific) before embedding. The blocks were stored at room temperature for four weeks prior to sectioning.

The tissue blocks were sectioned at 5 *μ*m thickness and placed on glass slides. The slides were dewaxed by placing them in a slide holder and taking them through xylene for 20 min with gentle agitation. The slides were then rehydrated by placing them in graded alcohol (100%, 100%, 90%, and 70% ethanol for 3 min each) and then gently in running tap water. The tissues were stained by placing them in Harris haematoxylin (Sigma Aldrich, Darmstadt, Germany) for 10 minutes. They were then washed slowly in running water and gently distained (using 1% HCl in 95% ethanol), rinsed again in running tap water for 5 min, then placed in Scott's tap water (bluing solution) (Leica Biosystems GmbH, Wetzlar, Germany) for 3 min, and then stained in eosin (Sigma Aldrich, Darmstadt, Germany) for 2 min. The tissues were then dehydrated in graded alcohol (70%, 90%, 100%, 100%, and 100% ethanol 5 h each) and then cleared in three changes of xylene for 5 min each and mounted using Permount mounting medium (Fisher Scientific, Fair Lawn, NJ, USA). Upon drying, the hematoxylin-eosin stained slides were examined under light microscopy.

Histological comparisons between samples from the three groups were made for structural tissue changes such as differentiation patterns, degree and type of keratinization, morphology and number of rete pegs, and connective tissue changes. Histological images were captured using the LASEZ Leica Application Suite EZ software under the Leica DM 750 microscope (Leica Microsystems GmbH, Wetzlar, Germany). Histomorphometric analysis of tissue sections was by computer based digital image analysis software (DinoCapture 2.0) using the DinoEye AM4023 digital eyepiece camera (AnMo Electronics Corporation, Taipei, Taiwan) connected to a computer via USB. Analysis of all tissue sections was done at similar settings at 200-fold magnification under an Olympus CX31 light microscope (Olympus Corporation, Tokyo, Japan).

**Figure 1:**
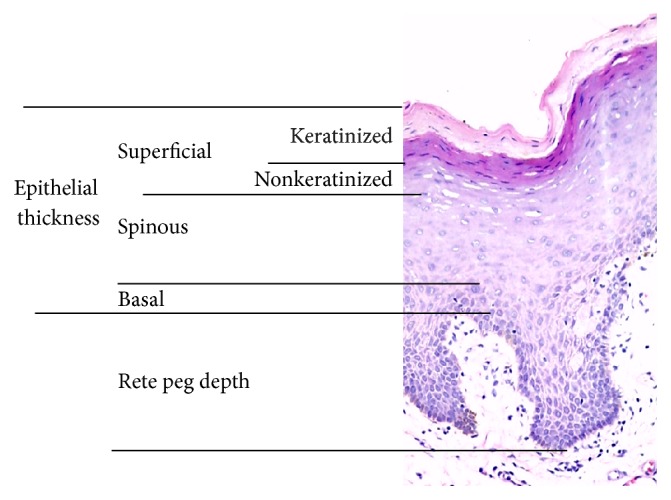
Histological preparation of one of the study samples at 200-fold magnification under light microscopy showing how the epithelium was divided into four main components for histomorphometric analysis. Thickness of the various sections was determined by drawing arbitrary lines running vertically and reading off the length/depth as was indicated by the software.

For comparative histomorphometry, the epithelium was divided into four main components: rete pegs, basal cell layer, spinous cell layer, and superficial cell layer (Figure 1). The superficial cell layer was further subdivided into keratinized and nonkeratinized components. Thickness of the various sections was determined by drawing arbitrary lines running vertically and reading off the length/depth as was indicated by the software. The layers were assessed for their thickness and compared with each other and with the total thickness of the epithelium.

### 2.4. Statistical Analysis

Data analysis and generation of figures were done using Sigma Plot software version 12.5 (Systat Software, Inc., San Jose, CA, USA). Data sets from the three groups were first subjected to Shapiro-Wilk normality test and then compared using one-way analysis of variance (ANOVA) or Kruskal Wallis Test. This was followed by multiple comparisons using either the Holm-Sidak or Dunn's methods as appropriate to determine the levels of significance between specific groups. *p* values less than 0.05 were considered significant. Data were presented as means ± standard error of the means.

## 3. Results

### 3.1. Characteristics of the Study Subjects

The control group of 8 volunteers had a mean age of 28.9 years (range 25 to 37). The first study group of 14 volunteers had a mean age of 33.6 years (range 21 to 52) whereas the second study group of 20 volunteers had a mean age of 39.8 years (range 25 to 55). All selected participants were chronic khat chewers who had used khat for more than 5 years. The only method of tobacco use reported was smoking. Seven (35%) of the smokers had smoked tobacco for less than ten years and the remainder (65%) had smoked for less than 10 years. Four (20%) of the smokers reported that they used 1 cigarette per day, 8 (40%) of them used between 1 and 10 cigarettes per day, 3 (15%) of them used between 1 and 10 cigarettes per day, and the rest (25%) of the smokers used over 20 cigarettes per day. In the analysis of age differences between the study groups, there was a statistically significant difference in the ages of the control group and the second study group composed of khat chewers who also smoked tobacco. Other than the two habits and the difference in the ages of the control group and the second study group, there were no other differences in health or behavioral characteristics in these groups.

### 3.2. Tissues from Khat Chewers Showed Abnormal Intracellular Edema and Keratinization

**Figure 2:**
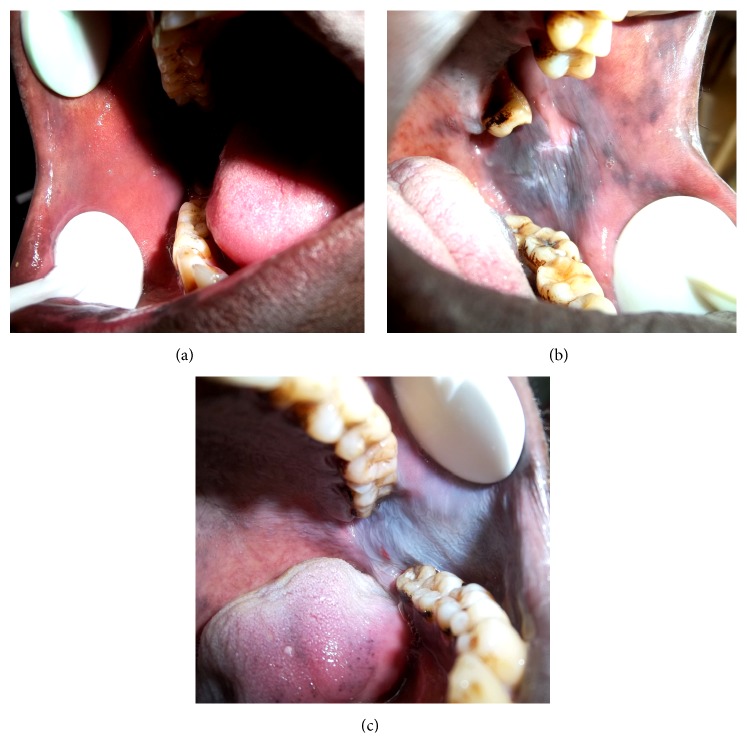
Clinical photographs of the buccal mucosa of some of the study participants. (a) Nonchewer (control), (b) a chronic khat chewer, and (c) a chronic khat chewer who was also a heavy smoker.

**Figure 3:**
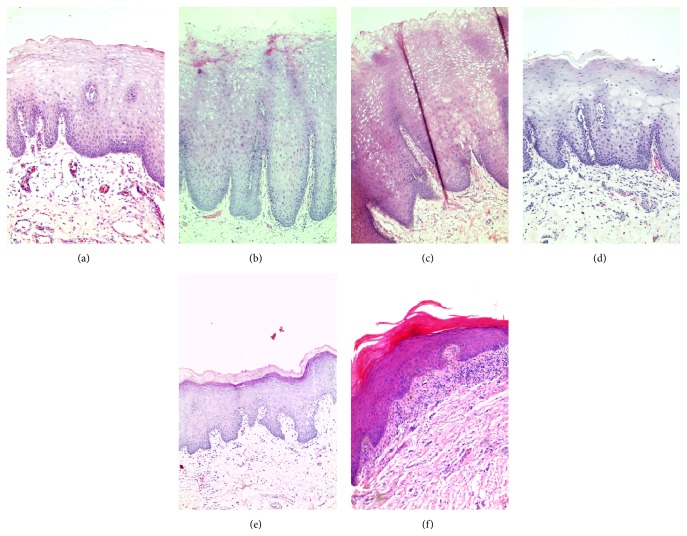
Histological preparations of biopsies from a nonchewer (a) and khat chewers. Acanthosis and intracellular edema are seen both in a smoker (b) and a nonsmoker (c). Varying degrees of keratinization are seen in samples from a mild khat chewer (d), a heavy khat chewer (e), and a heavy khat chewer who also smoked tobacco (magnification ×200). There were no histological differences between smokers and nonsmokers.

Clinically, khat induced lesions showed varied degrees of white and brown discoloration (Figure 2). Microscopic evaluation of the samples revealed marked acanthosis and an increase in cells showing intracellular edema in the spinous cell layer of tissue samples from khat chewers (Figures 3(b) and 3(c)) when compared to those from nonchewers (Figure 3(a)). Among khat chewers, 75% of those who smoked tobacco showed abnormal intracellular edema compared to only 14% in nonsmokers and 13% in controls (Table 2). Histopathological evaluation showed that superficial layers in samples from chronic khat chewers were keratinized while control samples were nonkeratinized. Only two of the eight samples (26%) from nonchewers showed a keratinized superficial cell layer compared to 71% of samples from khat chewers and 75% of samples from khat chewers who smoked tobacco (Table 2). The type of keratinization varied from parakeratinization (Figure 3(e)) in most samples to orthokeratinization in a few samples (Figure 3(f)). In some orthokeratinized samples, cells of the deeper section of the superficial cell layer contained keratohyalin granules and resembled the granular cell layer of skin tissue. Both types of keratinization were seen in chronic khat chewers as well as in chronic khat chewers who smoked tobacco with no differences noted in this regard between the two groups. Epithelial dysplasia was not seen in any of the biopsies studied.

### 3.3. Tissues from Khat Chewers Showed Increased Epithelial Thickness

**Figure 4:**
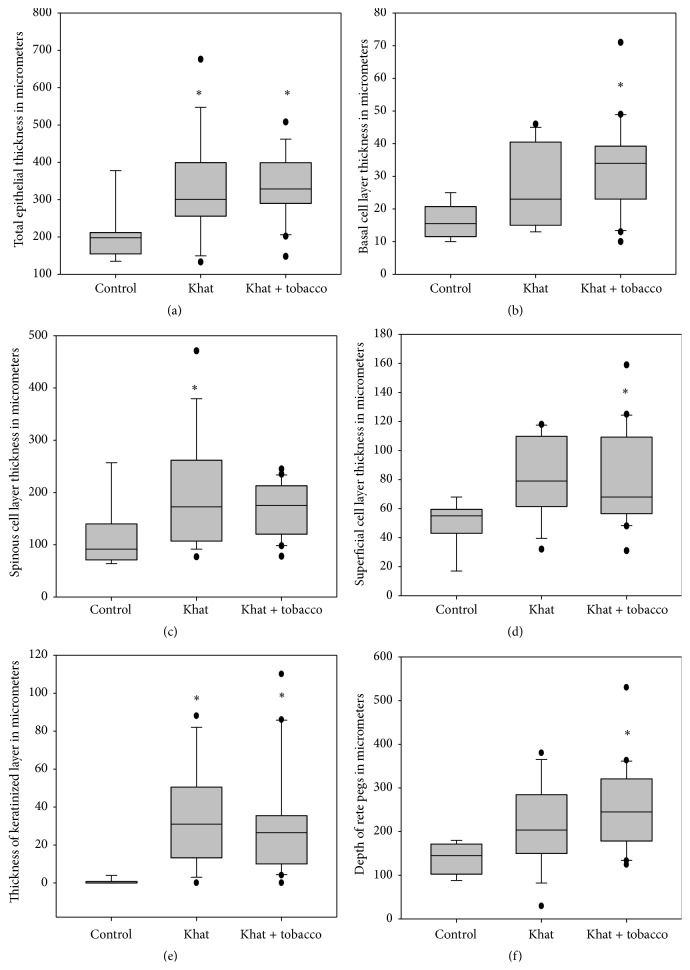
Analysis of the thickness of the buccal epithelium in controls and in study groups. Asterisk (*∗*) shows statistically significant differences (*p* < 0.05) between study groups when compared to the control after multiple comparisons.

Total epithelial thickness and thickness of various epithelial layers in samples from khat chewers were markedly increased when compared to samples from nonchewers (Figure 4). Epithelial thickness in control samples was found to be 205 ± 26 *μ*m whereas thickness in khat chewers and khat chewers who smoked tobacco was significantly higher measuring 330 ± 35 *μ*m and 335 ± 19 *μ*m, respectively (*p* < 0.05). The basal cell layer in control samples measured 16 ± 2 *μ*m whereas its thickness in khat chewers and khat chewers who smoked tobacco was significantly higher measuring 26 ± 3 *μ*m and 32 ± 3 *μ*m, respectively. The thickness of the spinous cell layer in nonchewers, khat chewers, and khat chewers who smoked tobacco was 113 ± 23 *μ*m, 195 ± 28 *μ*m, and 169 ± 11 *μ*m, respectively (*p* < 0.05). For the superficial cell layer, the thickness was 50 ± 5 *μ*m, 81 ± 8 *μ*m, and 79 ± 7 *μ*m in nonchewers, khat chewers, and khat chewers who smoked tobacco, respectively (*p* < 0.05). Even though the overall epithelial thickness and the thickness of constituent layers showed marked increase in tissues from khat chewers, the proportion of each layer relative to total epithelial thickness remained unchanged in all the groups measuring about 10%, 63%, and 27% for the basal, spinous, and superficial layers, respectively (Table 2).

The keratinized component of the superficial cell layer in nonchewers, khat chewers, and khat chewers who smoked tobacco was 1 ± 0.5 *μ*m, 34 ± 7 *μ*m, and 31 ± 6 *μ*m, respectively (*p* < 0.01) (Figure 4(e)). Whereas less than 1% of the superficial cell layer in control samples was keratinized, the proportion of superficial cell layer that was keratinized in khat chewers and khat chewers who smoked tobacco was 36.8% and 33.6%, respectively (*p* < 0.01). There was an increase in number of rete pegs per 100 *μ*m horizontally from 6.1 ± 0.4 in controls to 9.5 ± 0.8 and 7.8 ± 0.5 in khat chewers and khat chewers who smoked tobacco, respectively (*p* < 0.01). The rete pegs in khat chewers also appeared thinner and longer particularly among khat chewers who smoked tobacco. The differences noted between the groups with regard to number of abnormally shaped rete pegs were not significant (Table 2).

### 3.4. Abnormal Connective Tissue Changes in Tissues from Khat Chewers

**Figure 5:**
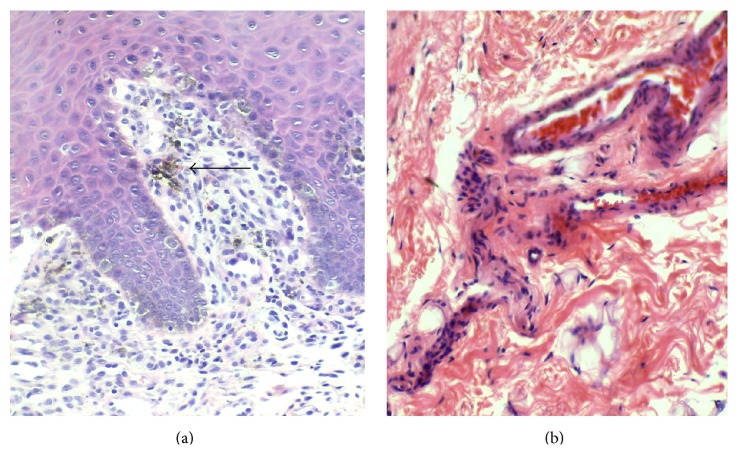
Connective tissue features in khat induced oral lesions. (a) Melanin pigment (arrow). (b) Fibrosis and tortuous blood vessels in the submucosa of chronic khat chewers.

Within the submucosa, an increase in the amount of collagen fibres (fibrosis) was noted in one control sample (13%), in four samples from khat chewers (29%), and in 7 samples among khat chewers who smoked tobacco (35%) (Table 1). Vessels within the submucosa of khat chewers were noted to be increased in number and were also more tortuous. There were deposits of melanin pigment and aggregates of melanophages scattered within the subepithelial area (Figure 5) and melanin was also seen within basal keratinocytes. A few samples from khat chewers also showed abnormal subepithelial infiltration of chronic inflammatory cells (Figure 3(f)) particularly lymphocytes. No differences were noted with regard to connective tissue changes between samples from khat chewers and khat chewers who smoked tobacco.

## 4. Discussion

This study compared histopathological changes in biopsies of the buccal mucosa from volunteers who have never chewed khat (controls), chronic khat chewers, and chronic khat chewers who smoked tobacco. Chronicity in khat chewing especially in relation to induction of oral keratotic lesions has previously been defined as a period of khat use exceeding 2 years as well as a high frequency of khat use, but not necessarily the amount of khat consumed per sitting [11, 14, 18]. Biopsies were taken from the most affected site on the buccal mucosa on clinical evaluation, which always coincided with the buccal mucosa on side of the mouth identified by the patient as the chewing side.

The buccal epithelium is normally nonkeratinizing, and this is consistent with what was observed in tissue samples from nonchewers in this study. However, epithelial tissues from khat chewers were found to be keratinized, with some tissues depicting orthokeratinization and formation of a layer similar to the granular cell layer of skin tissue. These findings are similar to those described in previous clinical studies [14, 18] and one laboratory study in organotypic models of oral mucosa [22]. According to our findings, less than 1% of the superficial cell layer in control samples was keratinized. However, the proportion of the superficial cell layer that was keratinized in khat chewers and khat chewers who smoked tobacco accounted for more than one-third of the thickness of that particular layer. To the best of our knowledge, no previous study has attempted to quantify in this manner the degree of keratinization in the buccal epithelium of chronic khat users in comparison to that from nonchewers. This study did not find any differences in the degree and type of keratinization between khat chewers and khat chewers who smoked tobacco, suggesting that concomitant use of tobacco has only a limited effect on overall appearance.

Normal thickness of epithelium of the oral mucosa varies depending on the site. The thickest epithelium is found in the buccal mucosa where it measures approximately 290 *μ*m [23]. In this study, epithelial thickness in khat chewers was significantly increased in general as well as in specific cell layers of the epithelium when compared to nonchewers. There were pronounced acanthosis and marked intracellular edema particularly in khat chewers who also smoked tobacco. These findings agree with those from a previous study that demonstrated acanthosis and intracellular edema as key features of khat induced mucosal changes [18]. However, contrary to what was observed in organotypic models of oral mucosa [22], the proportion of each of the layers relative to total thickness remained unchanged in all the groups and measured about 10%, 63%, and 27% for the basal, spinous, and superficial layers, respectively. The difference between our findings here and those described within* in vitro* organotypic models could be explained by the abundance of cells with stem-cell properties within* in vivo* epithelium that ensure enhanced compensatory responses leading to epithelial hyperplasia. This view is supported by the observed proportional increase in the thickness of the basal cell layer which suggests a higher capacity of the oral epithelium to replenish itself and to mount compensatory responses when compared to* in vitro* organotypic epithelium.

A point worth noting in this study is that no evidence of dysplasia was observed in any of the tissue samples studied. This is contrary to findings of a previous study [14] in which over 35% of the biopsies from the khat chewers who used tobacco were found to have dysplastic changes, particularly samples taken from the chewing side. Another study by the same author showed that the dysplastic changes were mild and the lesions could not be considered potentially malignant lesions [18]. Khat chewing has also been found to be a risk factor for developing cellular atypia as shown in a cytological assessment of buccal mucosa in chronic chewers [15]. In our view, the differences in the findings in these studies could be due to variations in chronicity of the khat chewing habit among participants in the different studies. The degree of tobacco use and the method of tobacco use among khat chewers in the different studies could also contribute to the observed differences.

Our study found a high concentration of melanin in basal keratinocytes and presence of scattered melanophages and melanin pigment in the submucosa of tissue samples of khat chewers. A higher proportion of tissues from khat chewers also showed fibrosis and many tortuous blood vessels as well as chronic inflammatory cell infiltrate. In this study, correlation between amounts of khat chewed and degree of pathological changes could not be determined due to the fact that we studied only one group (chronic khat chewers) and within group patterns were not detectable and were not presented in the results. There was also a limitation with regard to sample size due to the difficulty in finding volunteers willing to undergo biopsy procedures. There is a need for larger studies with four or more study groups of varying chronicity and khat consumption levels to enable correlation studies.

## 5. Conclusion

The findings of this study identify acanthosis, intracellular edema, hyperkeratosis, and fibrosis as key histological features of oral lesions induced by chronic chewing of khat. In addition, the study highlights key structural changes for specific layers of the epithelium and identifies increased melanin production as another factor contributing to the overall discoloration of the buccal mucosa in chronic khat chewers. From our findings, it appears that concomitant smoking has only limited effect on the clinical and histological appearance of khat induced oral lesions. As has been suggested before, and in view of the diverse histological and clinical presentation of these lesions, both physical and chemical factors are likely to play a role in their etiology.

## Figures and Tables

**Table 1:** Standardized criteria used to identify and to take biopsy of khat induced oral lesions.

Parameter	Criteria
Site of lesion	Normally limited to buccal mucosa where khat bolus is placed during chewing, around and especially below (inferior to) the *linea alba*. Most adjacent areas of buccal mucosa remain relatively normal and clearly distinguishable from oral white lesion. Could involve the lateral border of tongue and the gum. Usually does not involve the palate.

Texture	Lesion could be smooth, wrinkled, rough, or plaque-like.

Colour	Mostly white, but could be grey, black, or brown. A mixture of any of these colours could occur. Though rare, it could have red areas within it.

Side of the lesion	On the side identified as the chewing side.

Biopsy taking	Only from buccal mucosa. Biopsy an oval tissue of not more than 1 cm long and not less than 0.3 mm width. Preferably below the *linea alba*, or choose the centre of the lesion on buccal mucosa.

**Table 2:** Analysis of histological changes in the buccal mucosa.

	Nonchewers (*n* = 8)	Khat chewers (*n* = 34)		*p* value
		Nonsmokers (*n* = 14)	Smokers (*n* = 20)	
Samples with fibrosis (%)	13	29	35	—
Samples with intracellular edema (%)	13	14	75	—
Samples with keratinization (%)	26	71	95	—
Percentage of superficial layer keratinized	0.7 ± 0.3	36.8 ± 8.8	33.6 ± 6.2	*p* < 0.01
Number of rete pegs/100 *μ*m	6.1 ± 0.4	9.5 ± 0.8	7.8 ± 0.5	*p* < 0.01
Abnormally shaped rete pegs (%)	8.1 ± 2.8	9.8 ± 0.4	14.2 ± 2.4	*p* > 0.05
Rete peg depth as % of whole thickness	38.3 ± 3.0	34.8 ± 3.7	42.6 ± 2.7	*p* < 0.01
Proportion of basal cell layer (%)	8.9 ± 0.2	8.6 ± 0.5	11.4 ± 0.4	*p* > 0.05
Proportion of spinous cell layer (%)	61.3 ± 4.4	64.6 ± 4.6	60.4 ± 5.5	*p* > 0.05
Proportion of superficial cell layer (%)	27.9 ± 3.4	26.8 ± 6.3	28.2 ± 3.4	*p* > 0.05
